# Effects of mineral fertilization (NPK) on combined high temperature and ozone damage in rice

**DOI:** 10.1186/s12870-024-05695-0

**Published:** 2024-10-17

**Authors:** So-Hye Jo, Ju-Hee Kim, Ji-Hyeon Moon, Seo-Yeong Yang, Jae-Kyeong Baek, Yeong-Seo Song, Ji-Young Shon, Nam-Jin Chung, Hyeon-Seok Lee

**Affiliations:** 1 Crop Production & Physiology Division, National Institute of Crop Science, Rural Development Administration, Wanju-Gun, 55365 Republic of Korea; 2 Department of Crop Science and Biotechnology, Chonbuk National University, Jeonju, 54896 Republic of Korea

**Keywords:** Climate change, Combined stress, High temperature, Surface ozone, Rice

## Abstract

**Background:**

Increasing concern has recently been highlighted regarding crop damage due to extreme weather events caused by global warming and the increased production of ground-level ozone. Several studies have investigated rice growth in response to fertilization conditions under various environmental stress conditions; however, studies on growth development in response to fertilization conditions under combined high-temperature/ozone treatment conditions are scarce. In this study, we aimed investigate the growth and physiological development of rice under combined high temperature and ozone treatment conditions and to reveal the damage-mitigation effects of NPK fertilization treatments.

**Results:**

The plants were treated with varying levels of NPK [N2 (N-P-K: 9.0-4.5-4.0 kg/a), P2 (4.5-9.0-4.0 kg/a), K2 (4.5-4.5-8.0 kg/a), and control (4.5-4.5-4.0 kg/10a).] under combined high-temperature (35 ℃) and ozone (150 pb) treatment conditions. Analysis of the growth metrics, including plant height, leaf age, dry weight, and the plant height/leaf age (PH/L) ratio were revealed that combined high-temperature/ozone treatment promoted the phenological development indicated by increasing leaf age but decreased the plant height and dry weight indicating its negative effect on quantitative growth. The effects of this combined high-temperature/ozone treatment on growth were alleviated by NPK fertilization, particularly in K2 treatment but worsened in N2 treatment. Visible damage symptoms in rice leaves induced by exposure to the combined stressors was also alleviated by the K2 treatment. At the physiological level, K2 treatment reduced the expression of *OsF3H2*, which is associated with antioxidant activity, suggesting that potassium improved stress tolerance. Additionally, expression of genes related to abscisic acid (ABA) metabolism showed increased *OsNECD* (ABA synthesis) and decreased *OsCYP707A3* (ABA degradation) in the K2 treatment, promoting a stronger adaptive stress response. Stomatal conductance measurements indicated a slight increase under K2 treatment, reflecting enhanced regulation of stomatal function during stress.

**Conclusion:**

The study highlights the potential of potassium fertilization to mitigate combined high-temperature and ozone stress in rice, suggesting it as a strategy to improve crop resilience and optimize fertilization. The findings offer insights into fertilization treatments and can guide future research on stress tolerance in crops.

## Background

Global warming, driven by continued increases in greenhouse gas emissions, has intensified at an unprecedented rate over the past 50 years, particularly since the 1970s. This rapid climate change is projected to exacerbate complex and concurrent risks, including more frequent extreme heat waves and droughts [1]. Ground-level ozone, a secondary pollutant formed in conjunction with rising levels of air pollutants, volatile organic compounds (VOCs), and nitrogen oxides (NOx), severely affects crop productivity [2]. Several studies have underscored the negative effects of ozone on plants, such as reduced stomatal conductance, chlorophyll content, photosynthetic efficiency, and overall biomass [3–5].

Ozone formation is more pronounced on warm and sunny days with stagnant air. Therefore, changes in temperature, humidity, and UV radiation intensity due to climate changes are likely to affect the chemistry of ozone formation, which can consequently increase the ground-level ozone concentrations [6–8]. Studies have highlighted the strong association between elevated temperatures and ozone concentrations. Nevertheless, research on plant responses to these two stressors in combination or more complex environmental conditions is limited. Furthermore, the interactive effects of ozone and high temperatures on plants remain controversial and vary depending on the treatment conditions [9–11].

Rice is the staple food of approximately half of the world’s population and accounts for over 20% of the total calorie intake [12]. The normal growth and yield of rice relies on several factors, including the nutrient supply. The three major fertilizer elements, nitrogen (N), phosphorus (P), and potassium (K) play crucial roles in its normal growth. N is essential for protein synthesis that promote the development of grains and leaves, which are vital for overall plant health and productivity [13, 14]. P is a major component of ATP, facilitates energy transfer in essential processes, such as photosynthesis, protein synthesis, nutrient movement, and respiration, thereby influencing the crop growth and yield [15–17]). K significantly affects enzyme activation, protein synthesis, photosynthesis, and stomatal movement through turgor and osmotic regulation in plants [18] and plays a defensive role against abiotic stress [19]. These inorganic elements have interdependent relationships and exert diverse effects on crop physiology [20]. Therefore, understanding the interactions among these nutrients and their combined effects on stress resilience is essential for optimizing fertilization strategies under varying environmental conditions.

Previous studies have explored the effects of different fertilization conditions under high temperatures or ozone exposure on rice growth [21–23]. Given the frequent cooccurrence of these two stressors in nature, it is crucial to assess the effects NPK fertilization under the simultaneous stress of high temperatures and elevated ozone concentrations on rice productivity for developing effective management practices. We hypothesized that a multi-factorial experiment could reveal the interactive effects of different fertilization regimes under the complex environmental conditions due to climate change. To test this hypothesis, in this study, we aimed to investigate the growth development, oxidative stress, and related gene expression in rice subjected to combined high temperature and ozone gas treatment at planting time, focusing on the effects of NPK fertilization treatments. The findings of this study may offer valuable insights for developing strategies to enhance rice productivity and resilience under the compounded stressors induced by climate change.

## Methods

### Experimental site and materials

The experiments in this study were conducted in a growth chamber with controlled light intensity, temperature, and ozone gas concentration in an artificial weather facility at the National Institute of Crop Science in Jeonju, South Korea (35°49ʹ19ʺ N, 127°8ʹ56ʺ E). In this study, we used the rice variety, Samkwang (*Oryza sativa* ssp. japonica IT214880), which was provided by SeoYeong Yang (Rice production and Physiology Division, National Institute of Crop science). We obtained permission from the National Institute of Crop Science to use this variety https://www.nics.go.kr/api/spices.do?m=100000128&homepageSeCode=nics.

### Experimental design

To evaluate the effects of high temperature and ozone exposure on plant growth and physiological responses, a completely randomized design was employed to ensure unbiased distribution of plants across the treatment groups. All treatments were conducted under identical environmental conditions, except for the applied temperature and ozone levels. To ensure uniform growth, plants were cultivated for 14 days under controlled conditions, with an average temperature of 25 ℃ (MAX 30 ℃/ MIN 20 ℃), 14 h light/10 h dark, and 65% humidity. Fertilizer uptake was optimized by growing plants in 1/5000 Wagner pots for 14 days, with one plant per pot per fertilizer treatment. The fertilizer treatments were applied uniformly to each plant in 1/5000 Wagner pots according to the designated conditions for nitrogen (N), phosphorus (P), and potassium (K) levels in each treatment group. The fertilizer treatments included N2 (9.0-4.5-4.0 kg/a), P2 (4.5-9.0-4.0 kg/a), and K2 (4.5-4.5-8.0 kg/a), based on the control treatment (N-P-K: 4.5-4.5-4.0 kg/10a). The substrate used for plant cultivation was a loamy clay soil, well-suited for rice cultivation due to its balanced properties of drainage and nutrient retention. Fourteen days after transplanting, the plants were subjected to high temperature and ozone gas treatments (above mean air temperature and ozone) for 14 days. The optimal temperature for rice growth range from 25 °C to 30 °C (20 °C to 25 °C during the grain filling stage), with a critical temperature limit of 35 °C to 40 °C [24]. Accordingly, we set the temperature conditions at an average of 25 ℃ (highest 30 ℃, lowest 20 ℃) for the control group and 35 ℃ (highest 40 ℃, lowest 30 ℃) for the high-temperature treatment group for 14 days. Ozone was generated using an ozone generator CD-160 (Ambohr Electric, China) and monitored in real-time using an Ozone Monitor Model 465 L (Teledyne, USA). The plants in the control and high-ozone treatment groups were treated with 50 and 150 ppb ozone for 8 h a day from 12:00 to 20:00 over 14 days, respectively. The ozone treatment protocol was based on previous studies, which demonstrated growth differences after similar treatment durations [25, 26]. Plant development was monitored before and after treatment by conducting growth surveys, including dry weight, plant height, and leaf age. Additionally, the damage phenotype of the leaves and the expression patterns of stomatal conductance- and stress-response-related genes to determine the physiological responses to high-temperature and ozone exposure.

### Growth and visible damage on rice leaves

The fertilizer treatments were conducted under normal conditions and combined with high-temperature and ozone treatments. Both the control and treatment groups comprised 27 plants, each with 9 plants per N2, P2, and K2 treatments. Growth changes in response to the combined high-temperature and ozone treatments were observed before and after the treatment. Plant height was measured as the distance from the ground to the apex of the longest leaf. Leaf age was measured by counting the number of leaves unfolded from the main stem every 7 days, and the difference in development from the baseline before the combined high-temperature/ozone treatment was calculated. After the treatments, the above-ground plant parts were dried for 7 days in a 70 ℃ dry oven (OF-360 S; Jeio Tech Co., Ltd., Korea), and the dry weight was measured using a precision balance (FX-2000i; A&D, Korea). To determine quantitative growth, shoot dry weight was divided by the number of tillers. To determine the stress-induced visible damages to rice leaves, the number of damaged leaves due to high temperature and ozone exposure was counted at the whole leaf level after the combined high-temperature/ozone treatments. High-temperature damage in rice was identified as white burning at the tip of the leaf, and ozone damage was identified as chlorosis with brown spots on the leaf surface [27–29].

### Stomatal conductivity

Stomatal conductivity was measured using an SC-1 leaf porometer (Decagon Device, USA) under chamber conditions at approximately 10:00 am on the day the combined high-temperature/ozone treatments were completed. Before measurement, the desiccant was replaced and the device was equilibrated through a stability test. Subsequently, calibration was performed by placing a filter paper soaked in deionized water at the center of the calibration plate, repeated 5 to 6 times for accuracy. After calibration, stomatal conductivity was measured on two leaves per pot, excluding the flag leaf that was still developing at the time of measurement

### RNA extraction

On the day of treatment completion, the 2nd and 3rd leaves were collected from individual samples for analysis of ABA- and antioxidant-related gene expression. Rice leaves (0.1 g) were ground in liquid nitrogen. RNA was extracted using a Plant RNA extraction kit (Macherey-Nagel, USA) according to the manufacturer’s instructions. The principles of RNA extraction are described in [30].

### Quantitation real-time PCR

cDNA synthesis was performed on the purified RNA using the PrimeScript RT reagent kit with a gDNA eraser (Takara Bio Inc., Shiga, Japan). The synthesized cDNA was then analyzed using real-time PCR with SYBR^®^Green Realtime PCR Master Mix (Toyobo, Japan). All experiments were performed in triplicate. Ct values of each gene were calibrated and compared to the Ct value of the reference gene ubiquitin. The principles of real-time PCR are described in [30].

### Statistical analysis

All experiments were performed in triplicate, and the results are presented as mean values. Statistical analysis was performed using R software (version 4.0.3, R Foundation, Austria). Significant differences were assessed at *p* < 0.05 using a one-sample t-test and ANOVA, followed by Duncan’s multiple range test. Before statistical analyses, Levene’s test and the Shapiro-Wilk test were performed to assess the homogeneity of variance and normality of the data, respectively. When the assumptions of homogeneity or normality were not met, non-parametric tests, such as the Kruskal-Wallis test, were employed as they are more suitable for small sample sizes (*n* = 9). If the assumptions were met, parametric tests like ANOVA were applied. This approach allowed us to rigorously assess the effects of high temperature and ozone on plant growth and physiological responses, including dry weight, plant height, leaf age, stomatal conductance, and stress-related gene expression, both before and after the treatments.

## Results

### Effect of NPK fertilization treatment on rice growth before the combined high-temperature/ozone treatments

**Fig. 1:**
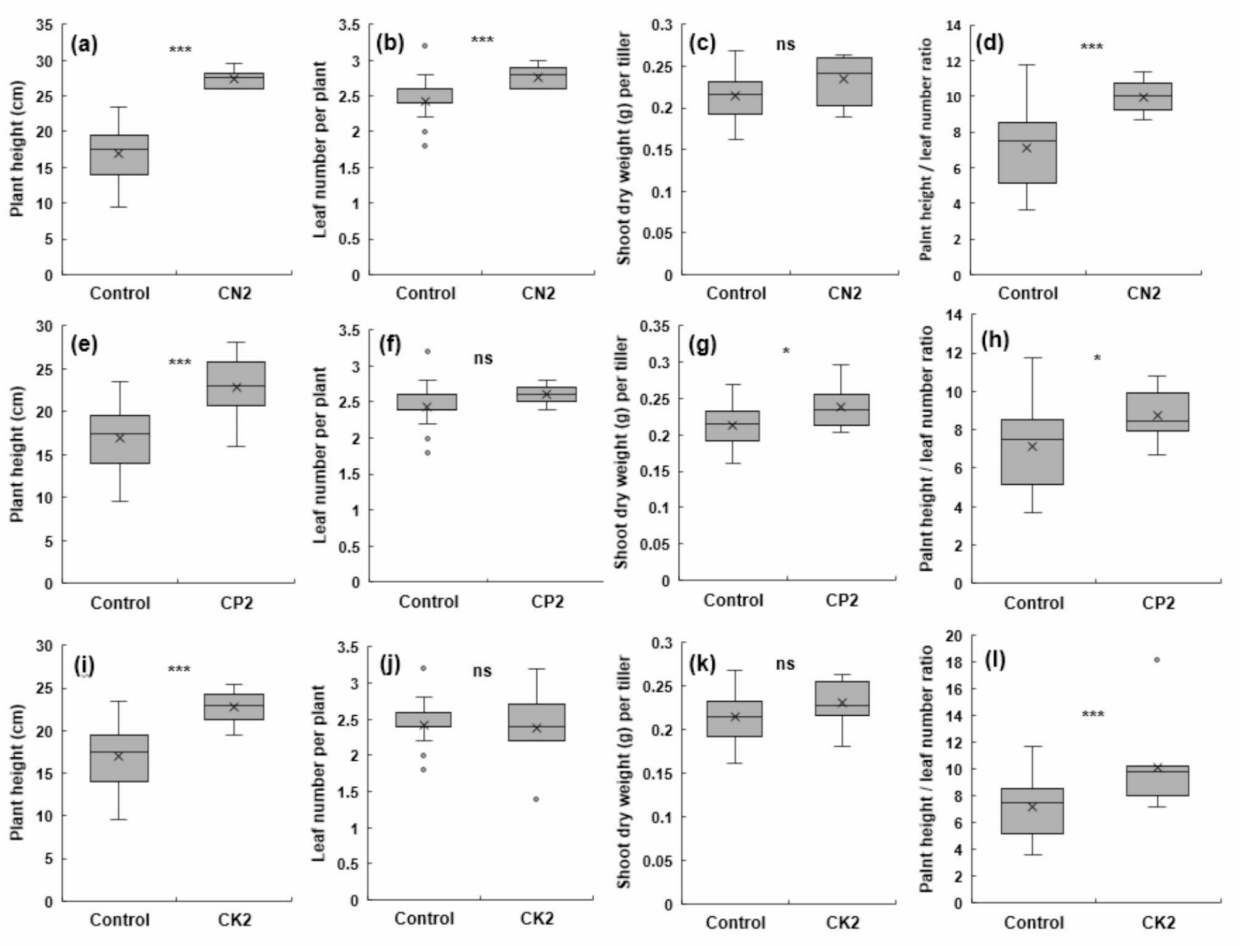
Effect of different NPK levels on rice seedling growths under normal conditions. Plant height **(a, e, i)**; number of leaves per plant **(b, f, j)**; shoot dry weight per tiller **(c, g, k)**; and plant height per leaf number **(d, h i)**. Three fertilization treatments were used: standard N, P, and K fertilization (control), N2 with standard P and K fertilization **(a-d)**, P2 with standard N and K fertilization **(e-h)**, and K2 with standard N and P fertilization **(i-l)**. Error bars represent the standard deviations, illustrating the variability within each treatment group. Asterisks denote statistical significance levels: ***p < 0.001, **p < 0.01, *p < 0.05, ns: not significant; Statistical significance was assessed using Student’s t-test comparing CN2/CP2/CK2 to the control group

We assessed the growth changes to evaluate the effects of NPK fertilization on rice before the combined high-temperature/ozone treatments (Fig. 1). Shoot dry weight and plant height were measured to assess the quantitative growth and leaf age was measured to determine phenological growth development according to fertilizer treatment. The changes in plant height, leaf age, dry weight, and the ratio of plant height to leaf age (PH/L) after two weeks of NPK fertilization before the combined high-temperature/ozone treatments are shown in Fig. 1a–k.

Plant height significantly increased by approximately 62%, 35%, and 35% in the N2, P2, and K2 treatments, respectively, compared with that in the control treatment (Fig. 1a, e, i). Leaf age significantly increased by 14% in the N2 treatment compared to that in the control treatment (Fig. 1b). In the P2 treatment, leaf age increased by 7%, while in the K2 treatment, it decreased by 2%; however, these differences were not significant (Fig. 1f, j). Dry weight also increased significantly by approximately 9%, 11%, and 8% for the N2, P2, and K2 treatments, respectively, compared to that in the control treatment (Fig. 1c, g, k), confirming that quantitative growth increased with increasing fertilization amount. The PH/L ratio increased significantly by approximately 40%, 23%, and 42% in the N2, P2, and K2 treatments, respectively compared to that in the control treatment (Fig. 1d, h, and i).

### Effect of the combined high-temperature/ozone treatments on rice growth

**Fig. 2:**
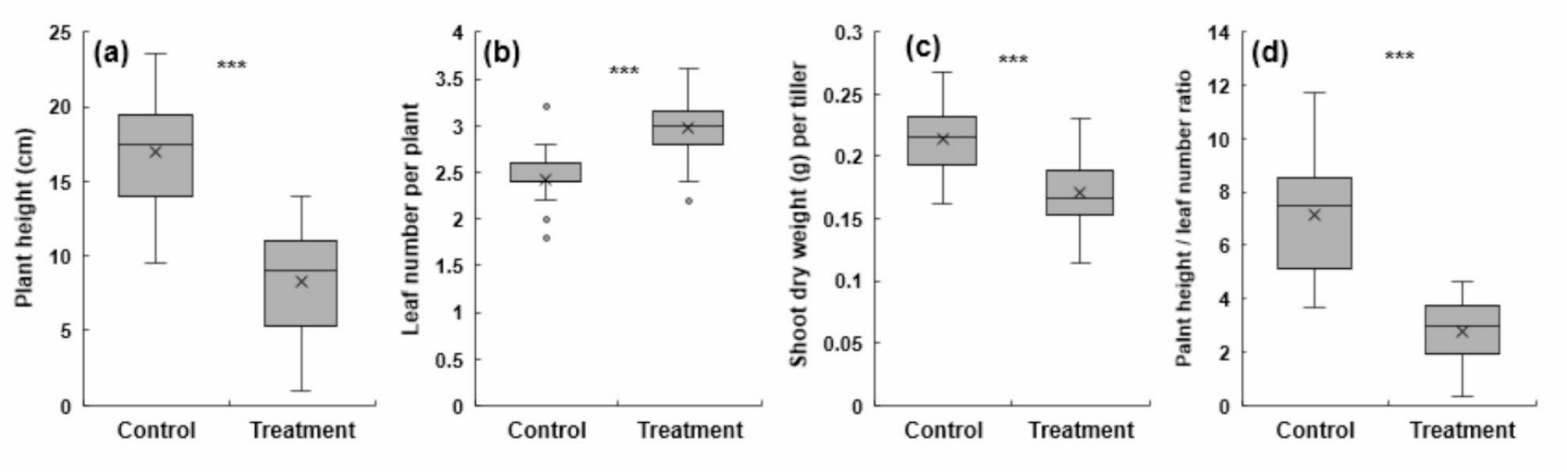
Changes in rice seedling growths in response to high temperature and elevated ozone treatment. height **(a)**, number of leaves per plant **(b)**, shoot dry weight per tiller **(c)**, and plant height per leaf number **(d)**. Student’s *t*-test was used to calculate P values. Asterisks denote statistical significance levels: ***p < 0.001, **p < 0.01, *p < 0.05, ns: not significant; significance tests were performed between Treatment vs. Control using Student’s *t*-test. Error bars represent the standard deviations

Next, we assessed the effects of combined high-temperature and ozone treatments on rice growth (Fig. 2a–d). Compared with normal conditions, leaf age increased by 23% under the combined high-temperature/ozone treatment conditions, indicating an accelerated leaf emergence rate (Fig. 2b). In contrast, quantitative growth decreased significantly after the treatment, with significant decreases in plant height (~ 51%) and dry weight (~ 20%) (Fig. 2a, c). The PH/L ratio was also reduced significantly to 61% in the combined high-temperature/ozone treatment condition compared to that in the control condition (Fig. 2d). These findings indicated significant reduction in quantitative growth compared to phenological growth development.

### Effect of NPK fertilization treatment on rice growth in the combined high-temperature/ozone treatment

**Fig. 3:**
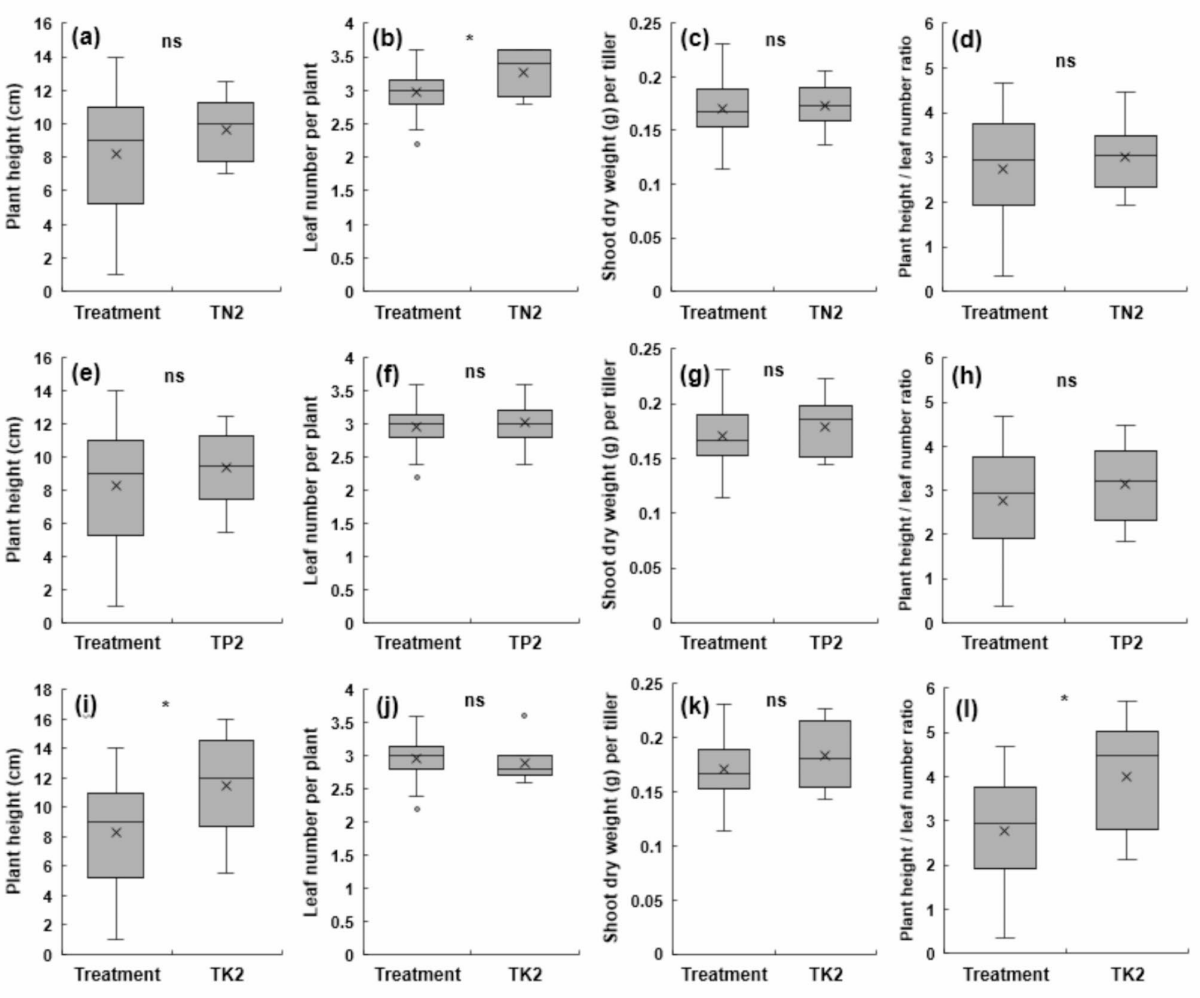
Effect of different NPK levels on rice seedling growths in high temperature and elevated ozone conditions. Plant height **(a, e, i)**; number of leaves per plant **(b, f, j)**; shoot dry weight per tiller **(c, g, k)**; and plant height per leaf number **(d, h i)**. Three fertilization treatments were used: standard N, P, and K fertilization (control), 2 N with standard P and K fertilization **(a-d)**, 2P with standard N and K fertilization **(e-h)**, and 2 K with standard N and P fertilization **(i-l)** Asterisks denote statistical significance levels: ***p < 0.001, **p < 0.01, *p < 0.05, ns: not significant; significance tests were performed between TN2/TP2/TK2 vs. Control using Student’s *t*-test. Error bars represent the standard deviations

The effects of NPK fertilization under the combined high temperature/ozone treatment conditions on plant height, leaf age, dry weight, and PH/L ratio are shown in Fig. 3a–k. Plant height significantly increased by approximately 39% from 8.2 to 11.4 cm in the K2 treatment compared to that in the control treatment under the combined high-temperature/ozone treatment conditions (Fig. 3i). The plant height also increased in the N2 and P2 treatments by 17% and 13%, respectively; however, the increase was not significant (Fig. 3a, e). For leaf age, the N2 treatment increased the rate of leaf emergence by approximately 10% compared to the control treatment (Fig. 3b). No difference was observed in the P2 treatment, and the K2 treatment showed a nonsignificant decrease by 2.6% compared to the control, indicating a slowing down of the leaf emergence rate in contrast to the N2 treatment (Fig. 3f, j). Dry weight was increased by 1.2%, 4.4%, and 7.1% in the N2, P2, and K2 treatments, respectively, compared to that in the control treatment; however, no significant difference was observed between the treatments under the combined high-temperature/ozone treatment conditions (Fig. 3c, g, k). The PH/L ratio did not differ significantly between N2 and P2 treatments compared to that in the control under the combined high-temperature/ozone conditions (Fig. 3d, h); however, it significantly increased by 44.9% in the K2 treatment (Fig. 3i).

### Symptoms of damage to leaves from combined high-temperature/ozone treatments

**Fig. 4:**
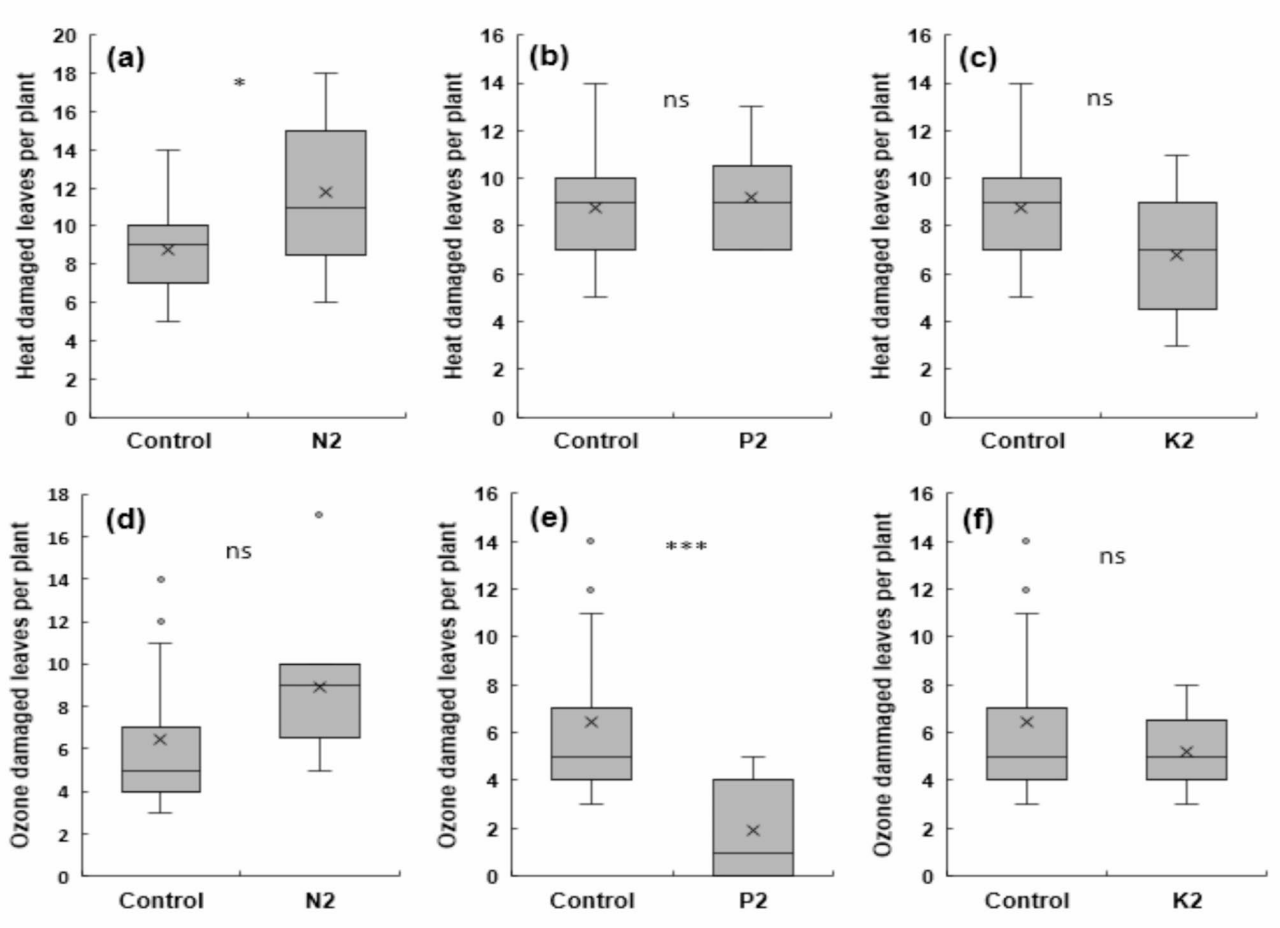
Effect of different NPK levels under high temperature and elevated ozone conditions on rice seedling leaves. Number of heat-damaged leaves per plant **(a-c)**; number of ozone-damaged leaves per plant **(d-f)**. Three fertilization treatments were used: standard N, P, and K fertilization (control), 2 N with standard P and K fertilization (a, d), 2P with standard N and K fertilization **(b, e)**, and 2 K with standard N and P fertilization **(c, f)**. Asterisks denote statistical significance levels: ***p < 0.001, **p < 0.01, *p < 0.05, ns: not significant; significance tests were performed between N2/P2/K2 vs. Control using Student’s *t*-test. Error bars represent the standard deviations

The effects of NPK fertilization on leaf damage under the combined high-temperature/ozone treatment conditions are shown in Fig. 4a–f. The number of damaged leaves by high temperature and ozone in the N2 treatment increased by 34% and 38%, respectively, compared with that in the control treatment (Fig. 4a, d). In P2 treatment, no consistent trend for high-temperature-induced damage was observed; nevertheless, the number of ozone-damaged leaves was significantly reduced by > 70% (Fig. 4b, e). The K2 treatment showed a 23% and 18% reduction in the number of high-temperature and ozone-damaged leaves, respectively, compared to the control (Fig. 4c, f), indicating that it mitigated both high-temperature and ozone-gas damage.

### Changes in gene expression related to antioxidant stress resistance and ABA pathway metabolism

**Fig. 5:**
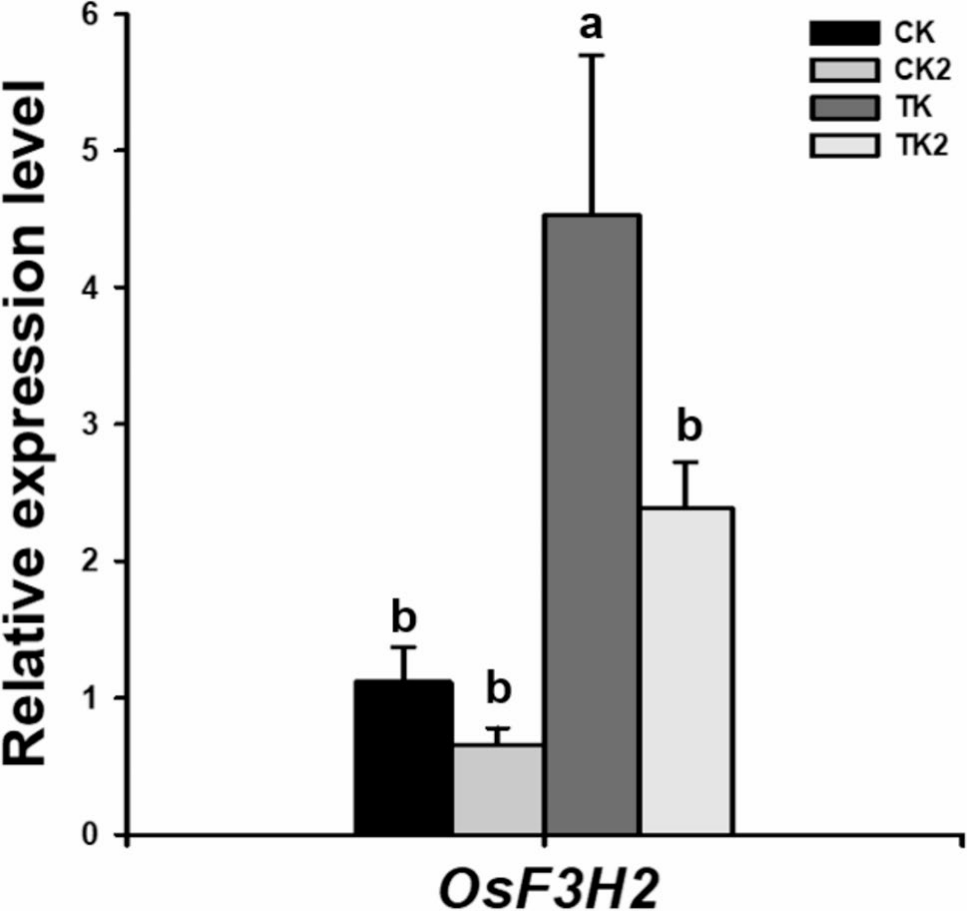
Changes in the expression of anthocyanin biosynthesis-related gene (*OsF3H*) in response to K2 treatment under high temperature and elevated ozone conditions. Significant differences were evaluated using Duncan’s multiple range test at the 5% level. Within each column, values followed by different letters **(a to b)** are significantly different (*p* < 0.05). Error bars represent standard deviations

**Fig. 6:**
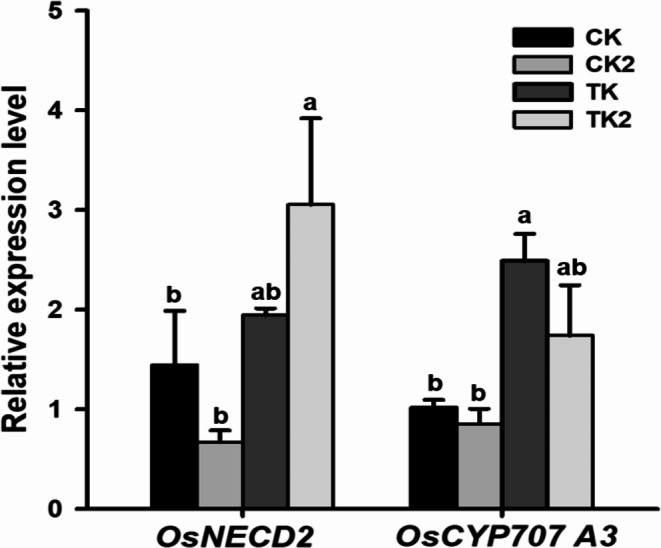
Changes in the expression of the abscisic acid synthesis-related gene (*OsNECD*) and ABA catabolism-related gene (*OsCYP707A3*) in response to K2 treatment under high temperature and elevated ozone conditions. Significant differences were evaluated using Duncan’s multiple range test at the 5% level. Within each column, values followed by different letters **(a to b)** are significantly different (*p* < 0.05). Error bars represent standard deviations

Next we assessed the effects of fertilization treatments under combined high-temperature/ozone conditions on the expression of genes associated with ozone resistance and abiotic stress using qRT-PCR. We focused on K2 treatment based on its effects on the growth and leaf damage of Samgwang variety in the presence of both the stressors. The expression of *OsF3H2*, an ozone resistance marker [1, 31] increased by > 5-fold under combined high-temperature/ozone conditions compared to that under normal conditions, confirming the increased antioxidant response to combined stress (Fig. 5). In contrast, K2 treatment alleviated the combined stressor-induced increases in *OsF3H2* expression (Fig. 5). *9-CIS-EPOXYCAROTENOID DIOXYGENASE (OsNECD)* gene promotes the synthesis of abscisic acid (ABA), a major abiotic stress-related phytohormone. In this study, the expression of *OsNECD* was elevated, indicating increased ABA synthesis (Fig. 6). In contrast, the expression of *OsCYP707A3*, which is involved in ABA degradation was decreased in the K2 treatment compared to that in the control under combined high-temperature/ozone treatment conditions (Fig. 6).

### Changes in stomatal conductance

**Fig. 7:**
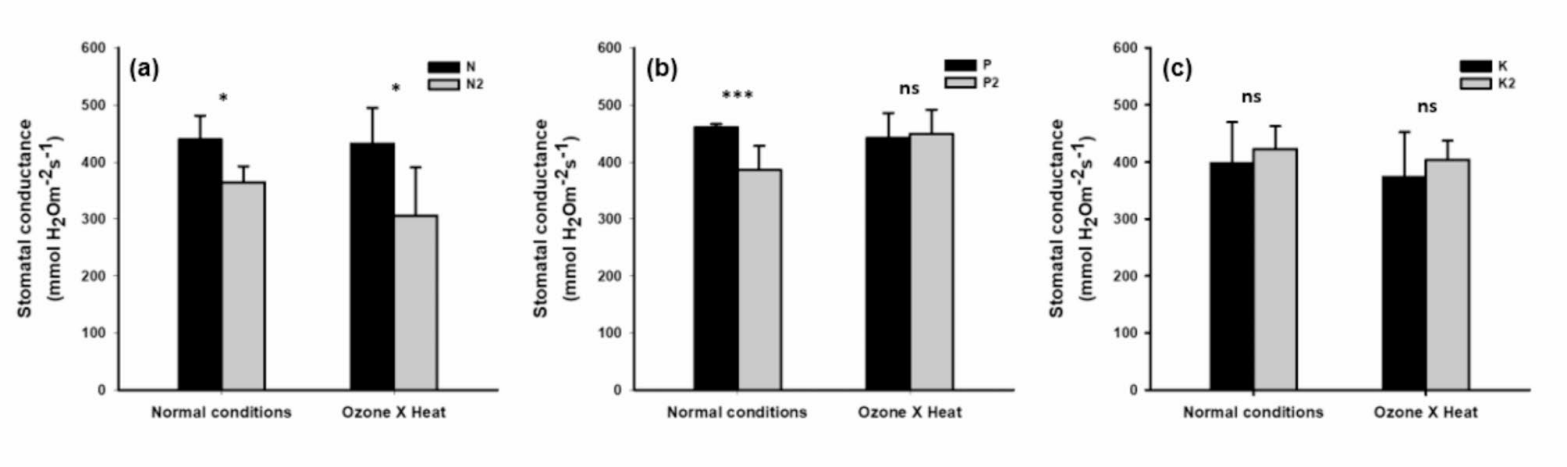
Effect of different NPK levels on stomatal conductance under high temperature and elevated ozone conditions. Three fertilization treatments were used: standard N, P, and K fertilization (control); N2 with standard P and K fertilization **(a)**; P2 with standard N and K fertilization **(b)**; and K2 with standard N and P fertilization **(c)** Asterisks denote statistical significance levels: ***p < 0.001, **p < 0.01, *p < 0.05, ns: not significant; significance tests were performed between N2/P2/K2 vs. Control using Student’s *t*-test. Error bars represent standard deviations

Figure 7 shows the effects of control and N2, P2, and K2 treatments under combined high-temperature/ozone conditions on stomatal conductivity. The results showed a significant decrease in stomatal conductivity by 17.2% and 29% in the N2 treatment compared to that in the control treatment under normal and combined high-temperature/ozone conditions, respectively (Fig. 7a). The P2 treatment significantly decreased the stomatal conductivity compared to the control under normal conditions, but did not show any difference under the combined high temperature and ozone conditions (Fig. 7b). Conversely, the K2 treatment increased stomatal conductivity by 6.2% and 8.2% under normal and combined high-temperature/ozone treatment conditions, respectively, although the difference was not statistically significant (Fig. 7c).

## Discussion

### Effect of K fertilization on rice growth under combined high-temperature/ozone stress

**Fig. 8:**
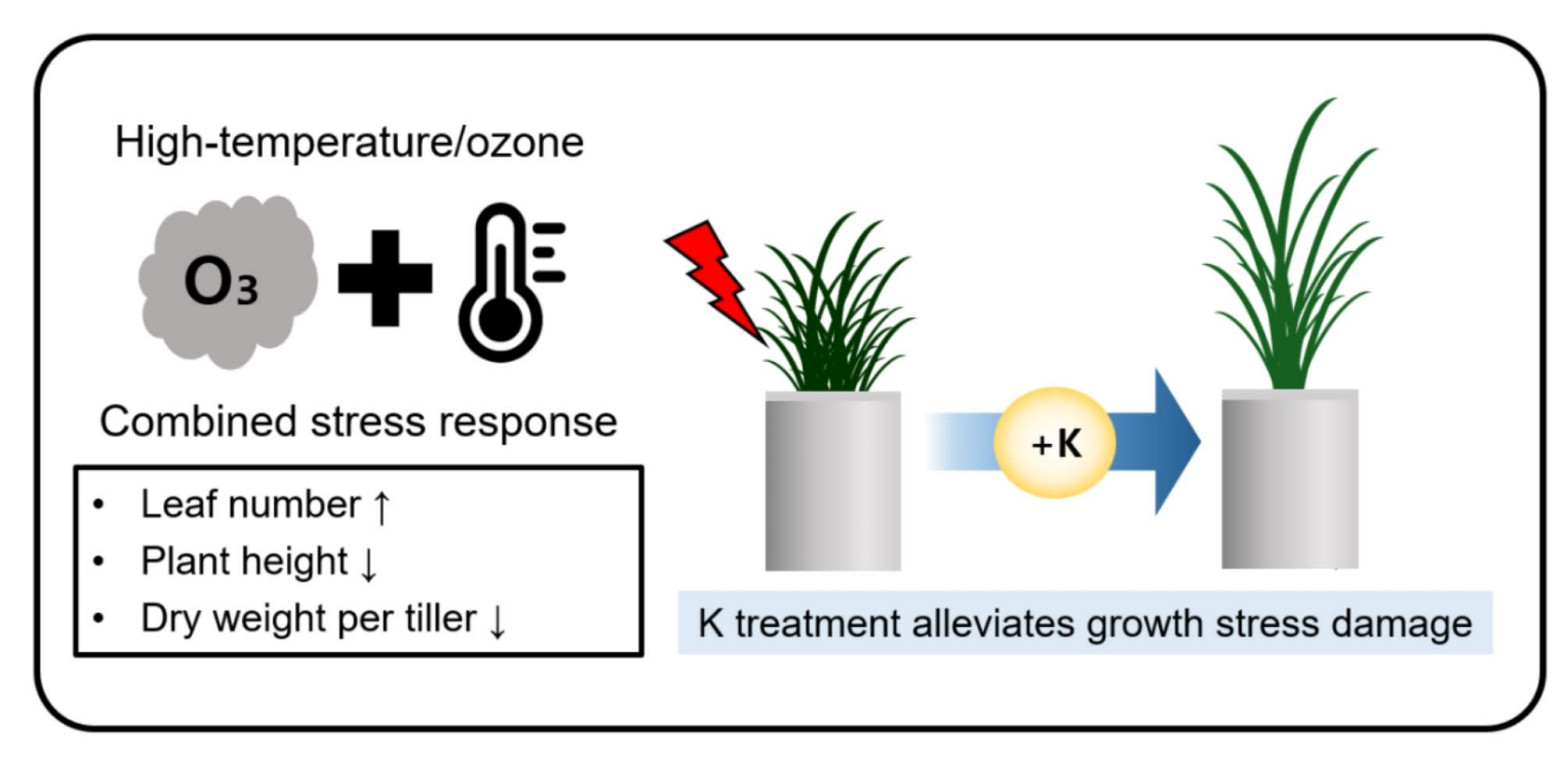
Schematic diagram of major growth changes in rice under combined high-temperature/ozone stress and the relief of stress damage when treated with K fertilizer

The rate of leaf development accelerates as temperatures rise, marking the transition from the vegetative to reproductive phases of the plant [32, 33]. Leaves play a critical role in photosynthesis and the transport of assimilated products to roots and spikelets. When quantitative growth, such as leaf size and weight, does not keep pace with leaf development, overall growth may suffer [34]. Given that leaf development and quantitative growth can respond differently under various stress conditions, this study evaluated the growth damage in rice plants subjected to combined high-temperature and ozone stress (Fig. 2). The findings showed an increase in leaf age under combined stress compared to normal conditions, likely due to the accelerated leaf development and phase transition induced by the stresses (Fig. 2). In contrast, plant height and dry weight, which are the indicators of quantitative growth, decreased significantly relative to the rate of growth development (Figs. 2 and 8). This decline is speculated to affect later growth stages, potentially leading to a decrease in the number of panicles per tiller and, consequently, yield.

Various studies on fertilizer management have sought to mitigate the damage caused by various environmental stress conditions due to climate change. NPK fertilizers, essential for growth, promote growth and boost yields when applied appropriately. Rice utilizes nitrogen (N) to synthesize proteins that promote grain and leaf development; however, excessive nitrogen can cause late-season grain deterioration. Wang et al. [21] demonstrated that higher concentration of nitrogen exacerbate growth damage under high-temperature stress un *Agrostis stolonifera* L., suggesting over-fertilization worsens stress damage. In contrast, another study reported that the nitrogen treatment can reduce the stress damage caused by high temperatures and elevated CO_2_ concentrations in *Capsicum annuum L.* [35]. These studies indicate that the effects of effect of nitrogen treatments varies by plant and stress type. In the present study, both plant height and leaf age increased with N2 treatment compared to control treatment under normal conditions (Fig. 1); however, under the combined high-temperature/ozone treatment conditions, N2 treatment did not significantly affect plant height and dry weight compared to the control (Fig. 3). Instead, only leaf age significantly increased, potentially amplifying the stress-induced damage. These findings are consistent with those of previous studies demonstrating that nitrogen treatment can exacerbate ozone-induced yield reduction in rice [23]. The combined impact of nitrogen over-fertilization and high-temperature/ozone stress may have worsened the growth damage in this study, underscoring the complex interactions between multiple stresses [36, 37]. Therefore, fertilization strategies need to account for various environmental factors, not just high temperatures.

In contrast, the K2 treatment significantly increased the PH/L ratio under combined stress, suggesting that potassium helped mitigate the damage. Potassium is known to reduce reactive oxygen species (ROS) production by inhibiting NADPH oxidase activity and enhancing stress tolerance by regulating osmotic pressure [19]. In this study, K2 fertilization appeared to enhance tolerance to combined stress and improve quantitative growth relative to development rate. Together, these findings suggest that nitrogen over-fertilization accelerates leaf development, hastening phase transitions and exacerbating damage under combined high-temperature/ozone stress, while K2 treatment alleviates these damages.

Abiotic stress affects plant growth and development, particularly the leaf surface, where it impairs photosynthesis. Among environmental stresses, ozone causes pale leaf color and dark brown spots, while high temperatures lead to dark green leaves and leaf tip dieback in rice [27–29]. In this study, high-temperature- and ozone-induced damage to leaves increased in the N2 treatment and decreased in the K2 treatment, consistent with growth patterns. Potassium plays a key role in stomatal regulation and osmotic balance. Excess nitrogen, which lowers the K/N ratio, can lead to chlorosis, thin leaves, and dark green leaf coloration [38]. These results suggest that nitrogen over-fertilization impairs potassium uptake, weakening resistance of the plants to abiotic stress. On the contrary, increased K fertilization appears to improve resistance to multiple environmental stresses. To further explore these findings, we examined stomatal conductance, ABA metabolism, and the expression of antioxidant-related genes.

### Physiological analysis of K fertilization to mitigate combined high-temperature/ozone stress

We investigated key physiological factors to better understand how potassium mitigates damage under combined high-temperature/ozone stress conditions. First, we confirmed that K2 treatment reduced damage caused by these combined stressors based on the growth data, and then examined expression of stress-related genes in K2-treated plants. In plants, flavonoids act as signaling molecules, UV filters, and ROS scavengers, helping plants cope with environmental stress [39, 40]. *F3H* is involved in anthocyanin biosynthesis and part of the flavonoid pigment family, enhances antioxidant capacity by increasing enzyme activity and gene expression in response to UV-B and cold stress [41]. The expression of *OsF3H2*, a member of the *OsF3H* gene family, is known to increase significantly under ozone stress and serves as a marker for ozone damage [1, 31]. In this study, *OsF3H2* expression significantly increased under combined high-temperature/ozone treatment conditions compared to that under normal conditions, likely due to higher anthocyanin accumulation and antioxidant activity under stress. However, K2 treatment reduced the high-temperature/ozone-induced increased expression of *OsF3H2* that had been elevated under stress conditions, suggesting that potassium enhanced the stress tolerance of the plants.

We also compared the expression of genes associated with the synthesis and degradation of ABA, a key hormone involved in stress tolerance. Zhang et al. [42] found that the expression of *OsNCED* rises rapidly in response to high-temperature stress, in rice seedlings, which decreases ROS levels and boosts antioxidant enzyme activity, thereby increasing heat stress tolerance in rice. Similarly, in *Arabidopsis*, high-temperature stress increases the expression of *AtNCED3* and decreases that of *AtCYP707A*, resulting in higher ABA content [43]. These findings consistent with those of previous studies indicated that plants adapt to abiotic stress by regulating ABA metabolism.

Previous studies on stomatal responses to high temperatures or ozone exposure have demonstrated that plants increase transpiration by opening their stomata at elevated temperatures to regulate internal temperatures [9]. In contrast, stomatal conductance decreases in response to ozone exposure [44]. In this study, we measured stomatal conductance to assess how rice plants regulate stomatal behavior under the dual stress of high temperature and ozone, and how various fertilization treatments affect stomatal movement. Our findings showed a decrease in stomatal conductance with N2 treatment, but a slight increase with K2 treatment. Higher potassium concentrations in plant cells cause stomata to open by increasing water absorption and cell swelling, which regulates evapotranspiration under stress [45]. Therefore, potassium deficiency impairs stomatal regulation, making plants more vulnerable to abiotic stress. Excess nitrogen in plants requires higher potassium levels, so nitrogen over-fertilization can lead to potassium deficiency [46], as seen in theN2 treatment, potentially reducing K uptake and thereby lowering stomatal conductance.

However, the relationship between stomatal conductance and ABA metabolism gene expression under K fertilization does not align with established theories. Generally, under abiotic stress, plants are expected to close their stomata by promoting K^+^ ion efflux from guard cells, driven by elevated ABA concentrations [47]. However, in this study, K2 treatment led to a slight increase in stomatal conductance, despite the expectation that it would decrease due to increased ABA synthesis gene expression. This suggests that stomatal regulation may not be solely driven by enzymatic processes but influenced by complex factors, such as changes in hormone levels under multi-stress conditions. Therefore, further research is necessary to better understand the mechanisms governing stomatal regulation in these complex environments.

## Conclusion

This study is the first to demonstrate the damage-mitigating effects of potassium fertilization in response to the combined stress of high temperature and ozone. This study confirmed the possibility of reducing combined high-temperature/ozone damage by fertilization control and is expected to be used as reference data for related research. To refine these findings, studies should explore the growth patterns across different fertilization concentration and develop damage-reducing cultivation methods by analyzing stress-related response indicators. Overall, these findings suggest the potential of potassium fertilization to reduce the effects of combined high-temperature and ozone stress on crops.

## Data Availability

All data and analyses are presented in the manuscript or Supporting Information. The source data for Figs. 1, 2, 3, 4, 5, 6 and 8 are provided as Source Data files.

## Abbreviations

ABA: Abscisic acid
ROS: Reactive oxygen species
NCED: 9-cis-epoxycarotenoid dioxygenase
NPK: Nitrogen Phosphorus, Potassium chemical fertilizer
PCR: Polymerase chain reaction
